# Case Report: Upper limb superficial venous thrombosis associated with oral contraceptives mimicking soft tissue mass

**DOI:** 10.3389/fcvm.2023.1263662

**Published:** 2023-11-13

**Authors:** Jun Hyeong Song, Kyoung-Chul Chun, Gwan Hee Han, Seung-Woo Yang, Sang-Hee Yoon, Jin-Sung Yuk, Jaeki Ahn, Sang Hyun Nam, Jongwoo Kim, Myounghwan Kim

**Affiliations:** ^1^ Department of Rehabilitation Medicine, Sanggye Paik Hospital, School of Medicine, Inje University, Seoul, Republic of Korea; ^2^ Department of Obstetrics and Gynecology, Inje University College of Medicine, Ilsan-Paik Hospital, Gyeonggi, Republic of Korea; ^3^ Department of Obstetrics and Gynecology, Sanggye Paik Hospital, School of Medicine, Inje University, Seoul, Republic of Korea; ^4^ Department of Plastic Surgery, Sanggye Paik Hospital, School of Medicine, Inje University, Seoul, Republic of Korea; ^5^ Department of Family Medicine, Sanggye Paik Hospital, School of Medicine, Inje University, Seoul, Republic of Korea

**Keywords:** forearm mass, forearm pain, oral contraceptives, upper extremity, venous forearm mass, venous thrombosis

## Abstract

**Background:**

Venous thrombosis associated with the use of oral contraceptives (OCs) occurs mostly in the deep veins of the lower extremity. A lesion of the upper extremity is rare, and the majority of thrombotic events that occur in the superficial vein of the upper extremity are caused by intravenous catheters. We present a rare case of superficial venous thrombus on the upper extremity in a woman with a history of long-term OC use.

**Case presentation:**

A 35-year-old woman, with an 8-year history of OC use, presented with a 2-year history of painfully palpable masses on her left forearm. The lesion mimicking soft tissue mass was confirmed to be superficial venous thrombi through ultrasound and magnetic resonance imaging. Conservative treatment including non-steroidal anti-inflammatory drugs, vasoprotective agents, and aspirin was prescribed. Through consultation with the Department of Obstetrics and Gynecology, it was confirmed that the current OCs could be discontinued, and the pain was almost relieved after conservative treatment.

**Conclusions:**

If thrombotic events occur in the superficial vein of the upper extremity without intravenous catheters, detailed medical history taking and the possibility of OCs should be considered.

## Introduction

A superficial soft tissue mass is a common indication for performing an imaging examination of the limb. Diseases of various pathologies, ranging from benign to malignant, appear as palpable masses or nodules in the skin. Soft tissue masses have different locations, and therefore, the location of the lesions sometimes aids in diagnosis. For example, epithelioid sarcoma can appear as single or multiple nodular lesions on the forearm, hand, and finger (1).

On the other hand, superficial venous thrombus (SVT) may also appear in the form of a palpable nodule and may be palpated like a cord depending on the length of the lesion. SVT occurs mainly in the lower extremities and is associated with immobilization, blood hypercoagulability, malignancy, and female hormonal therapies (2). In contrast, the prevalence of SVT in the upper limb is lower than that of SVT in the lower limb, and most of them are associated with intravenous catheterization (2, 3). SVT in the upper extremity after using oral contraceptives (OCs) is extremely rare, and only one case related to the brief use of contraceptives has been reported (4).

In this study, the authors report a rare case of SVT of the upper extremity that was misdiagnosed as soft tissue masses that occurred at an unusual site of the venous thrombus associated with long-term OC administration.

## Case report

A 35-year-old (gravida 0, para 0) female patient presented with a painful mass in her left forearm that first occurred 2 years ago. She stood 161 cm tall and weighed 54 kg. At that time, she first discovered a mass on her left forearm with a heating sensation, swelling, and pain. After she took a non-steroidal anti-inflammatory drug (NSAID), the size of the mass decreased and the pain was relieved. However, the pain recurred repeatedly, and an additional mass occurred in the proximal part of the existing mass 6 months before she visited our clinic. In addition, from that time on, she was prescribed an NSAID without any imaging study, and the pain continued without subsiding. She was referred to our clinic after visiting a primary medical institution, with suspected soft tissue masses on the left forearm.

**Figure 1:**
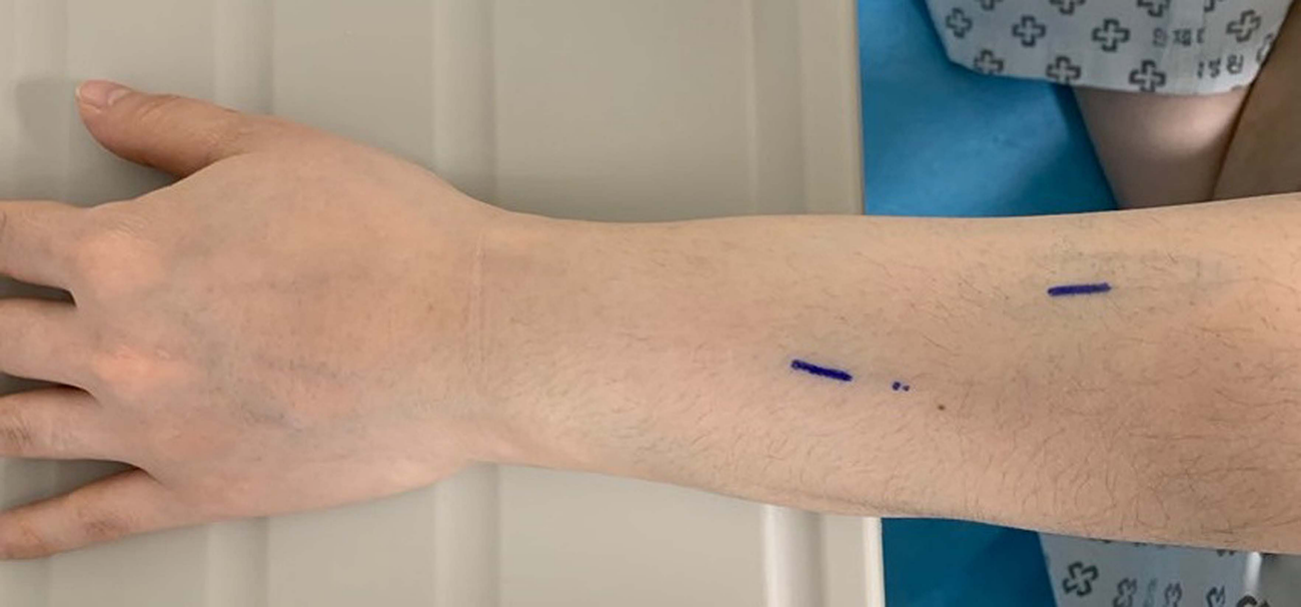
Appearance of the left forearm of the patient at presentation. Two linear masses are marked.

**Figure 2:**
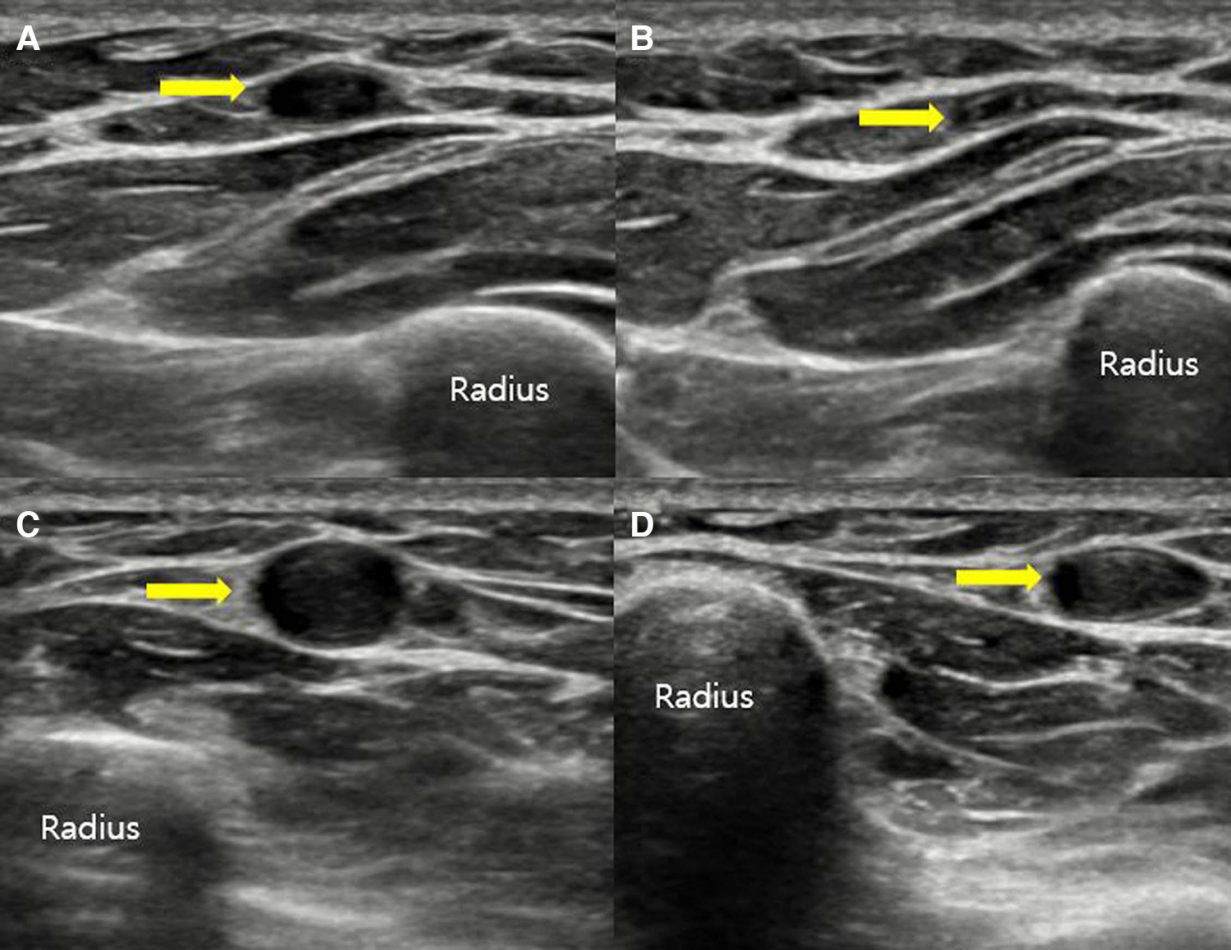
Ultrasound of the forearm in the short-axis view showing a normal compressible right cephalic vein (**A,B**) and a non-compressible left cephalic vein, indicating vein thrombosis (**C,D**).

**Figure 3:**
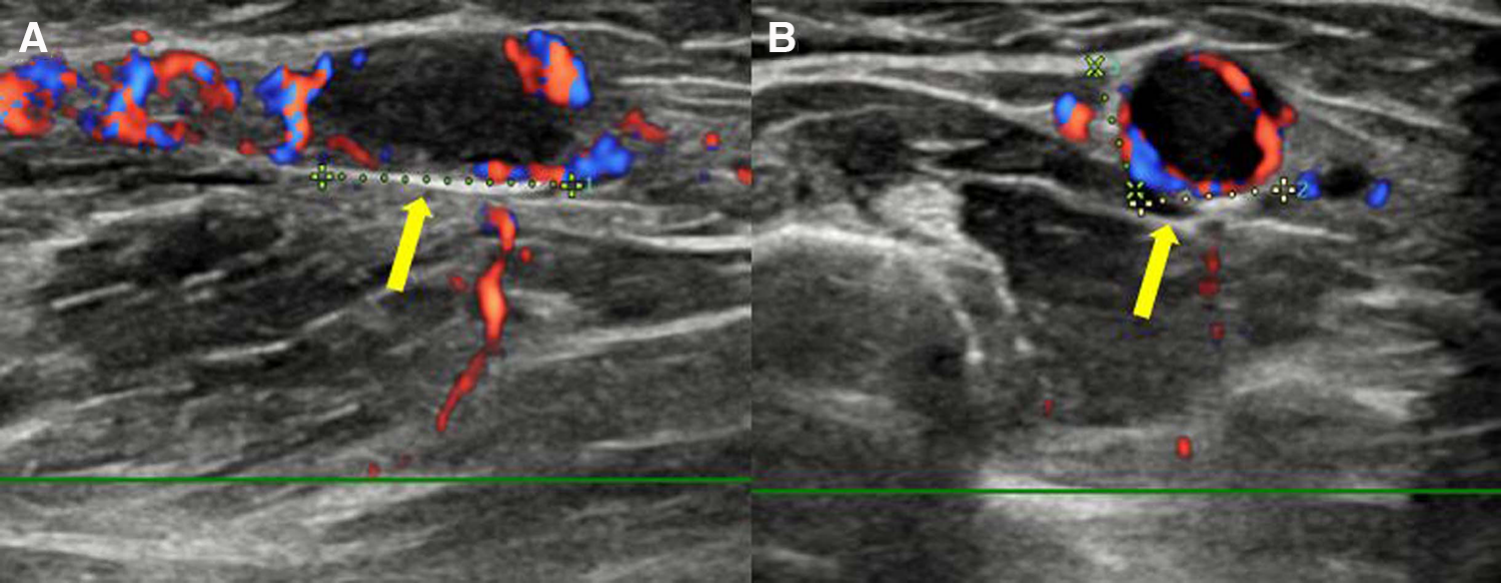
Doppler ultrasound of the left forearm shows an obstructive, ovoid, and hypoechoic mass in the long-axis view (**A**) and short-axis view (**B**). Thrombus (arrow) is filling the lumen of the cephalic vein, with the absence of a Doppler signal at its location.

**Figure 4:**
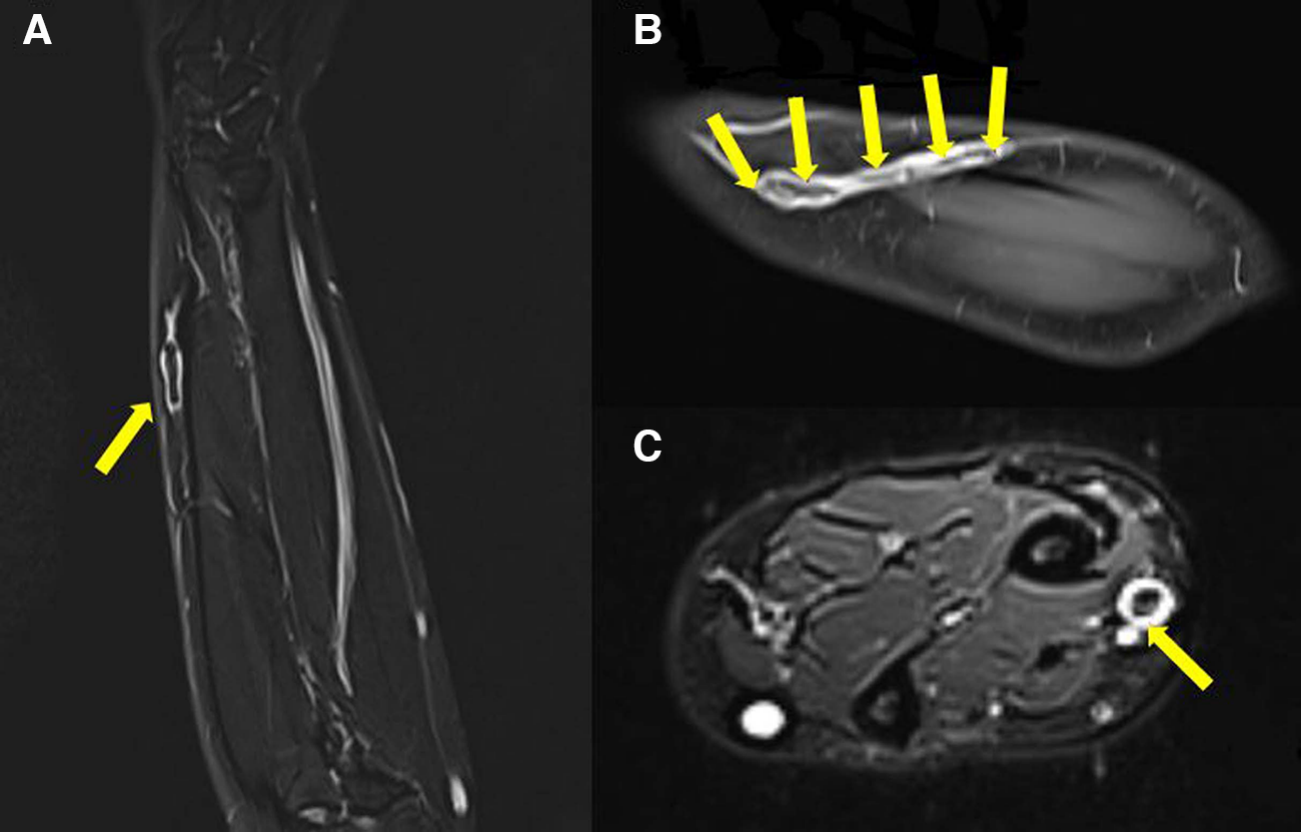
T2-weighted coronal section (**A**), T1-weighted coronal section (**B**) with contrast enhancement, and T2-weighted axial section (**C**) of the left forearm MRI. Non-enhancing low signal lesion (arrows) suspected to be thrombus located in the cephalic vein of the left forearm, extending 12 cm long.

Two linear masses were palpated in the longitudinal direction on the dorsal side of the left forearm of the patient (Figure 1). The masses with pain and tenderness were firm with clear boundaries. According to the patient, she experienced a heating sensation on both masses when the second mass first appeared. However, at the time of physical examination, the heating sensation of mass was absent. There were no fractures or soft tissue abnormalities on the plain radiograph of her left forearm. The long-axis and short-axis ultrasound confirmed an oval-shaped mass with a homogenous echogenicity filling the left cephalic vein (Figure 2). In the Doppler ultrasound, the vascularity of the masses was not increased, and the Doppler signal in the vascular lumen was absent. In addition, when the lesion was compressed with a probe, unlike the opposite forearm showing a normal collapse of the vein, there was no change in the shape of the vein, indicating thrombi in the cephalic vein (Figure 3). Furthermore, a contrast-enhanced magnetic resonance image (MRI) of the left forearm was performed to exclude the underlying risk factors of venous thromboembolism (i.e., malignancy). A 12-cm-long mass without enhancement, filling the cephalic vein in the longitudinal direction, was identified confirming SVT at the forearm, without any other soft tissue pathologies (Figure 4).

In an interview conducted after the imaging tests, the patient, who did not mention any special past medical history, reported that she had been repeatedly taking and discontinuing OCs (ethinyl estradiol 0.03 mg + drospirenone 3 mg) for 8 years due to endometriosis and that she had been taking OCs steadily for the last 2 years. Tumor marker, autoimmune antibody, and hematologic laboratory tests were performed to identify the risk factors of venous thrombus other than OCs. Protein C Ag level was 80% (70%–140%), and Protein S Ag level was 95% (70%–140%). The patient tested negative for anticardiolipin antibody, antiphospholipid antibody, and lupus anticoagulant antibody. AFP, CEA, CA 125, CA 15-3, and CA 19-9 levels were normal. The patient showed no history of immobilization of the left upper limb, smoking, air travel, local trauma, insect bite, or repeated local compression related to her professional or personal activities, and in recent years, there has been no instance of catheterization placed around the forearm lesion, such as intravascular injection or fluid therapy. In addition, no venous thrombi or varicose veins were observed in other parts of the body.

The patient was diagnosed with SVT in the left forearm caused by long-term use of OCs confirmed by the imaging tests, with the exclusion of other etiologies through medical history taking and blood tests. The patient did not complain of any respiratory symptoms, and fibrinogen degradation products (FDP) and D-dimer were in the normal range. No thrombus in the deep veins of the upper extremity was seen on the MRI, and computed tomography (CT) was not performed to confirm pulmonary embolism.

As part of conservative treatment, NSAIDs (zaltoprofen 80 mg 1 T t.i.d.) were administered, and local heat treatment was additionally performed as the inflammation was not in the acute phase. In addition, a vasoprotective agent (calcium dobesilate 1 T b.i.d.) was added after consultation with the Department of Vascular Surgery, and aspirin 100 mg 1 T q.d. was administered as part of prophylactic treatment for recurrent venous thromboembolism and major vascular events. Through consultation with the Department of Obstetrics and Gynecology, it was confirmed that the current OCs could be discontinued, and the pain mostly relieved after conservative treatment.

Three months later, the patient visited our clinic for a follow-up, and the ultrasound examination performed at that time showed that the blood clot remained in the vein. However, the pain relieved completely, following which the administration of anti-inflammatory drugs and vasoprotective drugs was discontinued. Aspirin was also discontinued after 3 months of administration. However, the patient experienced recurrent pelvic pain and vaginal bleeding because of endometriosis 6 months after discharge. After visiting a gynecologist, she was treated with a dienogest, an oral progestin that has a low risk of thrombosis. The pelvic pain relieved after the ingestion of dienogest.

## Discussion

OCs usually consist of estradiol (E2) or ethinylestradiol (EE), which increase procoagulatory factors to form a thrombus. In addition, depending on the type and administration method, progestin preparations incorporated into estrogen may promote or inhibit thrombus formation, and therefore, care should be taken in selection (5). For example, a dienogest, which was administered after the treatment of SVT in the present case, is a single-component progestin formulation that has minimal risk of clot formation when administered orally (5, 6).

SVT is a disease that has not yet been actively studied because its symptoms are mild and have a self-limiting course. Both superficial and deep vein thromboses are common in the lower extremities, and malignancies, autoimmune diseases, hypercoagulant conditions, and blood vessel damage increase the risk (3). On the other hand, SVT occurring in the upper extremities also shares the same risk factor but is caused by vascular damage related to procedures and treatments, such as injection of peripheral blood vessels and fluid therapy, in most cases (2, 7). In particular, SVT cases of the upper extremities related to long-term OC use are extremely rare, and only one case associated with brief OC use has been previously reported (4). SVT of the upper extremities in the previous case caused pulmonary embolism, and SVT associated with OCs, as in our case, is extremely rare.

The standard guidelines for the treatment of isolated SVT without deep vein thrombosis or pulmonary embolism are not clear (2). First, when it occurs in the lower extremities, the severity of the disease and the treatment vary depending on the size or location of the blood clot. In the case of small blood clots, which are usually defined as less than 4–5 cm in length on ultrasound, the prognosis is good, and local compression therapy using stockings, heat therapy, and medications such as NSAIDs are prescribed (2, 3). Conversely, thrombi larger than 4–5 cm are likely to lead to complications and require more active treatment such as anticoagulation. In addition, for SVT located within 3 cm of the saphenofemoral junction or saphenopopliteal junction, anticoagulant therapies such as warfarin, heparin, and new oral anticoagulants (NOACs) are provided as in cases of deep vein thrombosis (3, 8).

On the other hand, there is no approved treatment guideline for SVT in the upper and lower extremities (2). In the case of SVT caused by intravenous catheterization, the removal of the catheter is urgent, and if a catheter infection accompanies this condition, an antibiotic treatment appropriate for the causative agent can be given. To reduce pain and inflammation, conservative treatments such as topical heat treatment and the application of oral or gel-type anti-inflammatory drugs are mainly provided. In the case of isolated SVT in the upper limb without deep vein thrombosis, the effectiveness of anticoagulant therapy has not been proved (2, 3). Low-dose aspirin (100 mg/day) can be used as an alternative anticoagulant therapy even if aspirin cannot significantly reduce the rate of recurrence of venous thromboembolism, but this resulted in a significant reduction in the rate of major vascular events in the ASPIRE trial (9). In our case, the painful mass of the upper extremity of the patient responded to NSAID at first, but the subsequent mass did not. It is plausible that the first mass could have been attributed to a local inflammatory process stemming from various causes, which potentially contributed to the development of SVT.

As described above, the SVT of the upper extremities can be commonly seen in relation to the intravenous route of administration of drugs and fluids. However, in order to accurately diagnose the disease as in this case, it is necessary to consider not only clinically common situations but also rare situations, and accurate and detailed medical history taking is required.

We report a rare case of a patient with an SVT in the upper extremity, which was misdiagnosed as a soft tissue mass that occurred in an unusual site and was associated with OC administration without a catheterization history around the lesion.

## Data Availability

The original contributions presented in the study are included in the article/Supplementary Material, further inquiries can be directed to the corresponding author.
